# Intelligent computation in cancer gene therapy

**DOI:** 10.3389/fgene.2024.1252246

**Published:** 2024-03-14

**Authors:** Roee Samuel, Ramez Daniel

**Affiliations:** Department of Biomedical Engineering, Technion—Israel Institute of Technology, Haifa, Israel

**Keywords:** gene therapy, gene circuits, artificial neural networks, analog computation, digital computation

## Abstract

In recent years, the use of gene therapy for the treatment of disease has gained substantial interest, both in academic research and in the biomedical industry. Initial experimentation in gene therapy has generated positive results, as well as questions regarding safety. However, lessons have been learned from these first investigations, among them a realization that such treatments require a method to fine-tune the expression of therapeutic genes in real-time. A logical solution to this problem arose through the field of synthetic biology in the form of synthetic gene circuits. Thus, the synthetic biology community today aims to create “smart cells” for a variety of gene therapy applications, in an attempt to precisely target malignant cells while avoiding harming healthy ones. To generate safer and more effective gene therapies, new approaches with emerging computational abilities are necessary. In this review, we present several computational approaches which allow demonstrating artificial intelligence in living cells. Specifically, we will focus on implementing artificial neural networks using synthetic gene regulatory networks for cancer therapy and discuss the state-of-the-art computational developments.

## Introduction

Ever since the first U.S. Food and Drug Administration (FDA)-approved gene therapies came out in 2017 in the form of a treatment for the inherited eye disease Leber congenital amaurosis (FDA, 2017) and a chimeric antigen receptor (CAR) T cell therapy (NOVARITIS, 2019), the field of gene therapy has generated increasing interest in many medical fields. Although not the first gene therapies to be approved by a body of regulation for widespread use (Pearson et al., 2004; Cheever and Higano, 2011; Ylä-Herttuala, 2012; Milazzo et al., 2016), their approval launched forward subsequent gene therapies both in the same fields (MacKay et al., 2020; Prado et al., 2020) as well as in others. However, even with revolutionary results with certain malignancies, gene therapy treatments have been battling adverse side effects and sub-optimal effectivity since the outset (Bonifant et al., 2016; Schubert et al., 2021). The lack of control over the activity of gene therapies post-administration is an important problem which alludes to lacking safety, and so is a possible hindrance to their widespread adoption. For example, in the widely acclaimed CAR T therapy, adverse side effects may include cytokine release syndrome (CRS) and neurotoxicity which at times emerged in patients with fatal results (Brudno and Kochenderfer, 2016; Titov et al., 2018; Majzner and Mackall, 2019; Rubin et al., 2019).

In pursuance of a platform to solve current and future hurdles in gene therapy, a natural step forward was found in the field of synthetic biology. Synthetic biology is a multidisciplinary field of research that applies engineering principles to redesigning organisms for useful purposes. Thus, synthetic biology holds key aspects which allow it to demonstrate and develop the full potential of gene therapy. Control over timing, location, and degree of activity of genes, driven by certain forms of biological computation, have been well established in synthetic biological circuits in the last three decades (Becskei and Serrano, 2000; Elowitz and Leibler, 2000; Gardner et al., 2000; McAdams and Arkin, 2000; Hasty et al., 2002; Weiss et al., 2003; Basu et al., 2004; Friedland et al., 2009; Levskaya et al., 2009; Lu et al., 2009; Daniel et al., 2013; Walseng et al., 2017). Therefore, incorporation of synthetic biological circuits in current and future gene therapies, to create a real-time and precise control of genetic expression, is a likely answer to prevailing complications.

Since its onset, immunological (Muul et al., 2003) and oncological (Blaese et al., 1995) applications have led forward research in the field of gene therapy, adding novel tools to the newly created field of immunotherapy and extending opportunities in other medical fields with parallel applications. Therefore, although computation plays an important role in synthetic biology in medicine as a whole, in this review we focus on synthetic biological computation in biological cancer treatments—cell therapy, bacteriotherapy, and virotherapy.

## Biological computation

**FIGURE 1:**
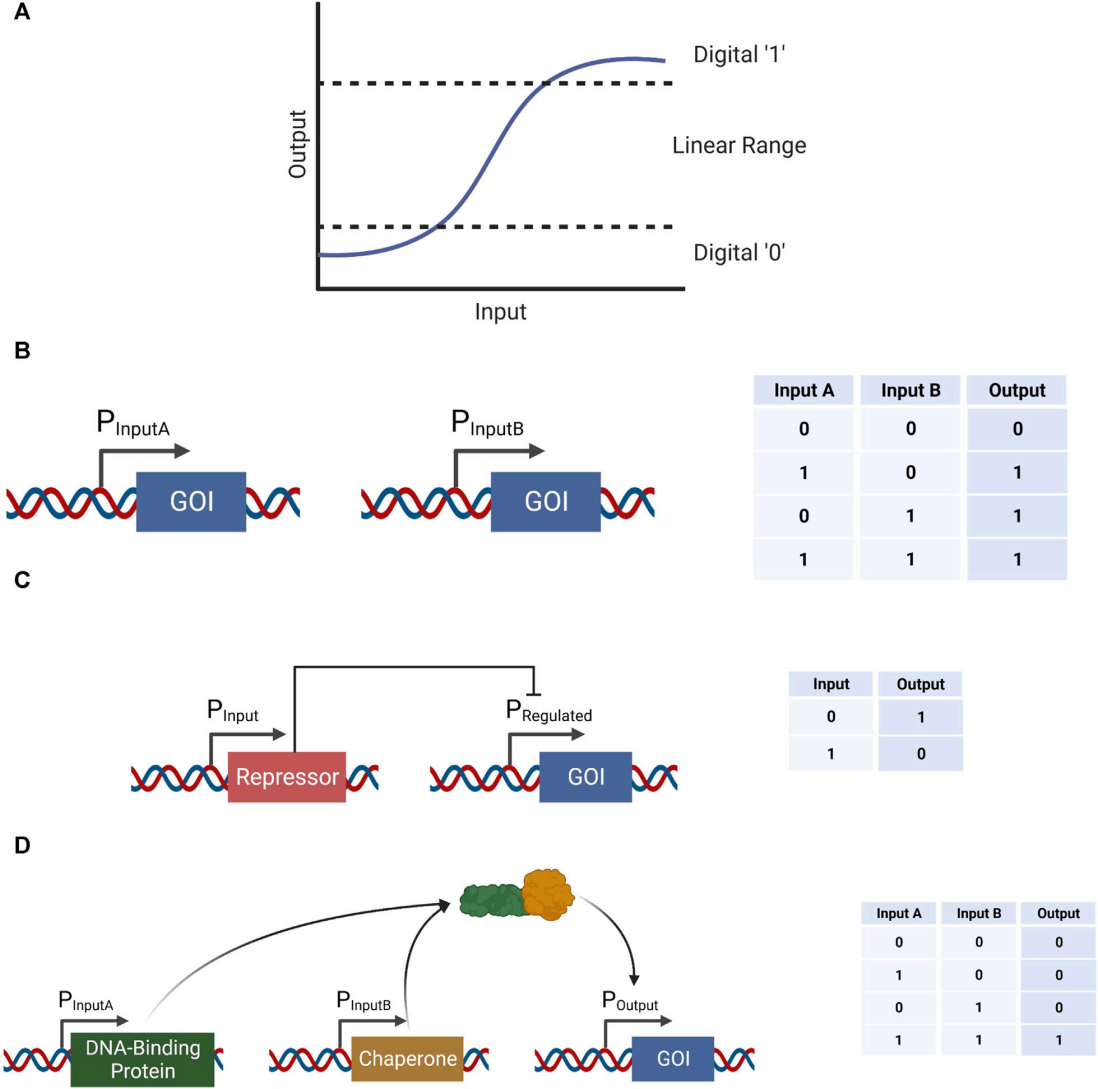
Example logic gates implemented in biological systems in various methods. **(A)** A conceptual representation of an input-output function and the corresponding digital “0” and “1” ranges, as well as the analog range in between. **(B)** An example of an OR gate using two separate promoters expressing the same gene of interest (GOI) in response to the presence of two different inputs. **(C)** An example of a NOT gate using a single input-activated promoter. This promoter expresses a repressor for a promoter that expresses the GOI, therefore increase in the input would result in a decrease in the output, and vice versa. **(D)** An example of an AND gate using two separate promoters expressing complementary proteins, which when combined are able to perform as an activator to a third promoter expressing the GOI.

Biological systems execute biological computations in order to detect internal or external stimuli, assess their implications, and carry out appropriate changes in behavior in response. To date, by far the most common form of computation implemented in synthetic genetic circuits in living cells is digital computation. This approach, which consists of computing with two discrete binary states (0 and 1), has proven useful in generating a variety of genetic tools (Figure 1A). Logic gates, including AND (Moon et al., 2012), OR, XOR (Siuti et al., 2013), NOT (Wang et al., 2011), NAND (Wang et al., 2011), NOR (Tamsir et al., 2011), and XNOR (Siuti et al., 2013), are an intuitive form of digital computation that have all been previously implemented in biological systems in various methods.

An OR gate can be implemented by using two different promoters each expressing a copy of the same output gene (Figure 1B). A NOT gate can be constructed by expressing a repressor in the presence of an input, which in turn represses the expression of a gene of interest (GOI) (Figure 1C). An AND gate, on the other hand, can be implemented by expressing two genes under two different promoters—each one activated under a different stimulation (Moon et al., 2012). These two proteins would have complementary purposes in activating a third promoter driving the expression of a GOI; one protein would have the ability to recognize and attach to a DNA sequence within the output promoter, and the second protein would be able to attach to the first protein, as well as attract an appropriate RNA polymerase to the transcription start site (Figure 1D). Logic gates can be implemented using metabolic and biochemical reactions as well (Barger et al., 2019). For example, the luxCDABE cassette was split into two parts, each part regulated by a different input. Thus, only when the two inputs were present the two parts were expressed, emitting a light. Other logic gates mentioned above can be constructed using an arrangement of the AND, OR, and NOT gates, and so their design would not be elaborated further.

**FIGURE 2:**
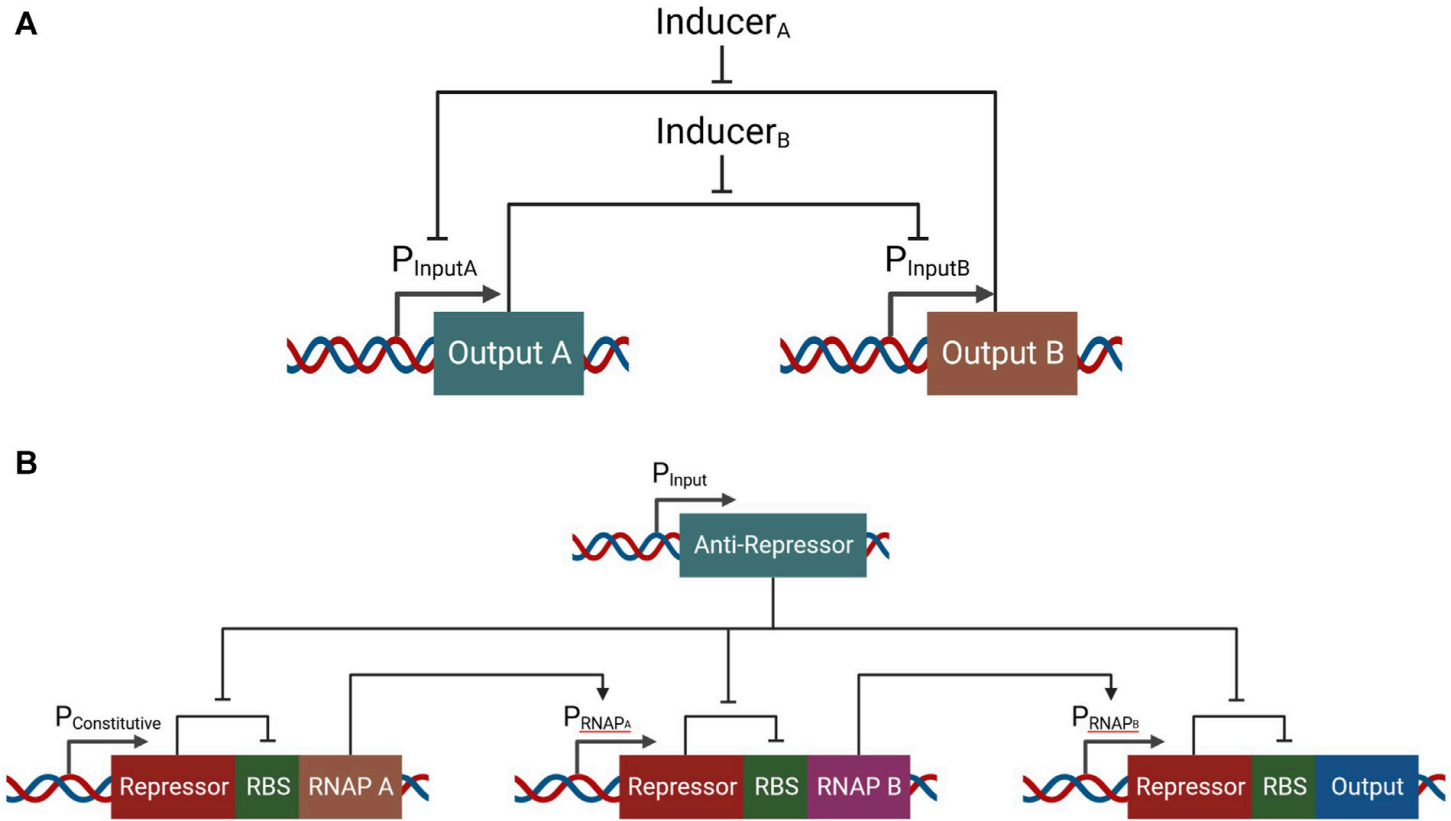
Example of a switch/memory system and a counter implemented in biological systems. **(A)** A mutually repressive gene system, with two inducers which can be used to change the state of the system, creating a new stable system. **(B)** A genetic system which responds with an output according to the number of times the system has been induced. In this genetic circuit, three sequential induces are required in order to generate a proper expression of the output gene.

Memory (Siuti et al., 2013), switches (Gardner et al., 2000), and counters (Friedland et al., 2009) all operate with digital information and have been implemented in biological systems as well (Figure 2). The toggle switch is an interesting example of both a biological switch and biological memory. As a control element that holds two mutually exclusive states, the toggle switch acts as a digital bit and provides information simply by being in one state and not the other. By expressing two (controllable) genes that repress the promoter of each other—generating a symmetrical mutual repression system—a toggle switch can be produced, resulting in either one of two states which remain stable over time, thus forming a binary biological state memory unit (Figure 2A). A counter, which counts up to three induction events, has been implemented through a ribo-regulated transcriptional cascade (Figure 2B).

**FIGURE 3:**
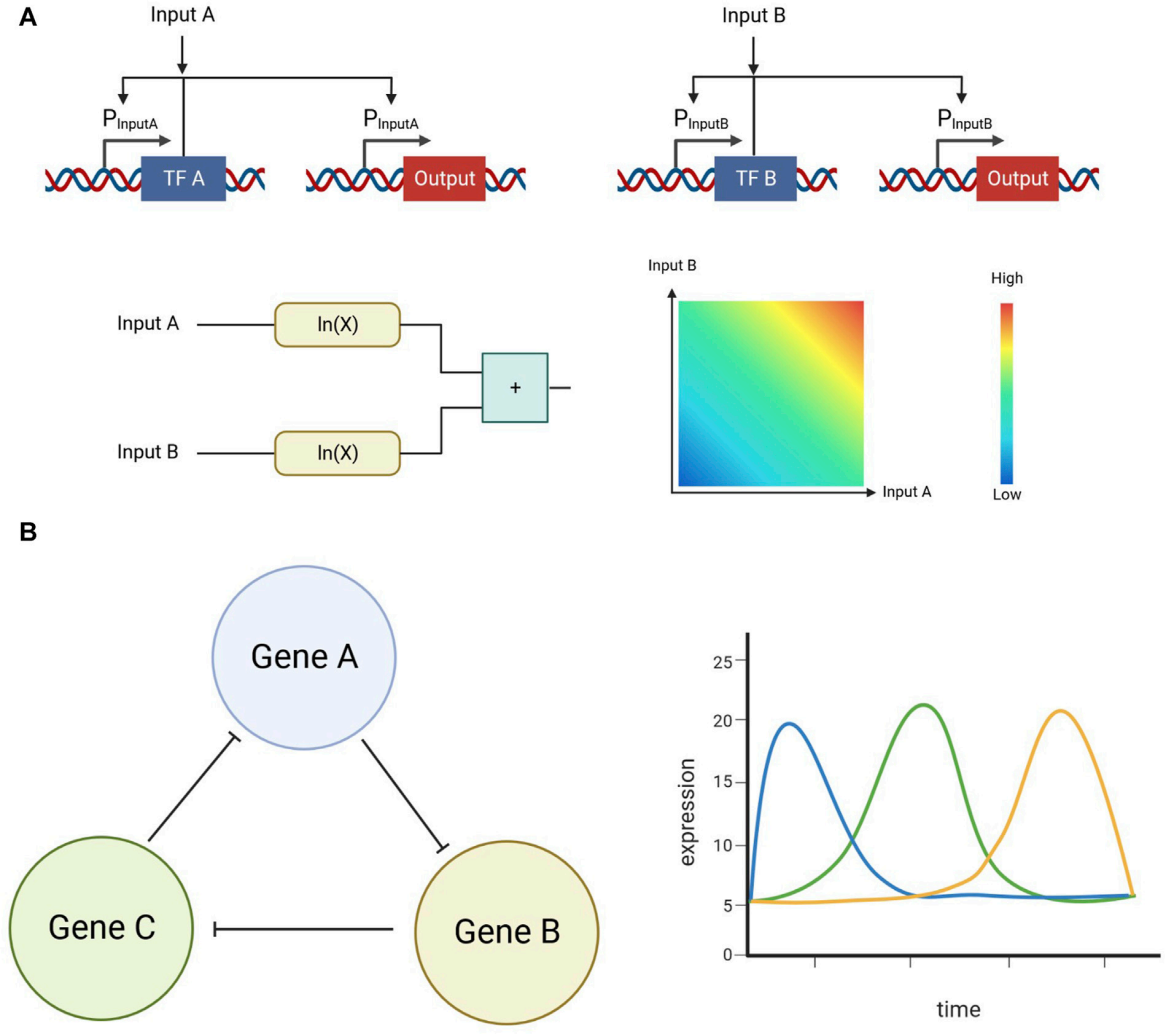
**(A)** Example of an analog addition operation implemented in biological systems. Positive feedback was used in these circuits to linearize the transform function, increasing the linear range on the inputs **(B)** Example of an oscillator implemented in biological systems. This system utilizes three repressors that repress the expression of each other in a symmetrical form.

As opposed to biological digital computation, biological analog computation excels at handling continuous inputs and outputs. Mathematical operations, which require continuous input and output domains, have been executed using biological analog computation in bacteria (Daniel et al., 2013); (Figure 3A). Since mathematical operations need the flexibility of continuous input and output ranges, biological analog computation is an effective way of implementing them. Driving stability, as well as instability, are another type of process which can take advantage of analog computation. For example, a robust adaptation system that drives population stability can be realized by adding an analog control system that acts as an integration feedback (Aoki et al., 2019). On the other hand, by generating a genetic circuit with three repressors which repress a different gene in the circuit, an oscillator can be realized (Elowitz and Leibler, 2000); (Figure 3B).

## Biological computation in current cancer gene therapies

Since logic gates could be used for classification of cells as malignant, current cancer gene therapies focus on the digital form of biological computation. Both immune and nonimmune cells have been utilized in research for the detection and elimination of cancer. One of the most explored gene therapies in cancer is CAR T cell therapy. This solution involves drawing blood from the patient, isolating T cells, engineering them to express a synthetic receptor, and reinfusing them into the patient. The chimeric antigen receptor is a chimera of intracellular elements of a T cell receptor and CD3ζ and the reshaped extracellular elements of a B cell receptor. Co-stimulatory elements are usually added to the chimeric antigen receptor, making 2nd generation (one co-stimulatory element) and 3rd generation (two co-stimulatory elements) CARs (Sadelain et al., 2013). By using an extracellular part taken from the B cell receptor, the synthetic receptor avoids the requirement for an antigen to be presented by a matching antigen-presenting molecule to initiate activation.

In search of an alternative platform for cancer treatment using synthetic biology in gene therapy, several researchers are turning to bacteria. The use of bacteria to cure cancer is not a new idea, with one of the first forms of cancer bacteriotherapy appearing at the end of the 19th century (Wiemann and Starnes, 1994). With modern medical technology granting safer circumstances for experimental exploration, a new interest in microbes is emerging, with a focus on synthetic biology’s designer microbes (Harimoto and Danino, 2019). As the most prevalent type of cell for experimental proof-of-concept synthetic biological circuits, the utilization of bacteria features a favorable platform for avoiding limitations that prevail in mammalian cell-implemented synthetic genetic circuits. Furthermore, cancer bacteriotherapy can better deliver therapeutics locally within the tumor microenvironment due to their tendency to localize to solid tumors (Lemmon et al., 1997). As such, it provides a platform with reduced toxicity associated with systemic drug delivery and allows a more closely controlled dosage.

Finally, Oncolytic viruses (OVs) are both naturally occurring and engineered viruses that infect and kill cancer cells, where the lysis of the tumor occurs directly by the activity of the virus or indirectly with assistance from immune cells. The interest in viruses for cancer treatment started following clinical reports of cancer regression coincidental with natural virus infections through the first half of the twentieth century (Kelly and Russell, 2007). Viral infection can naturally induce activation of the immune system and creation of local inflammation, which when happens at the site of a tumor at the hands of OVs can have positive anti-tumoral effects (Achard et al., 2018). Furthermore, by releasing tumor-associated antigens upon oncolysis to the immune-aroused microenvironment, OV activity can result in effect in a tumoral vaccination, unintentionally resulting in regression of related yet uninfected tumors (Bommareddy et al., 2018; Russell and Barber, 2018).

## AND gate in cancer gene therapies

**FIGURE 4:**
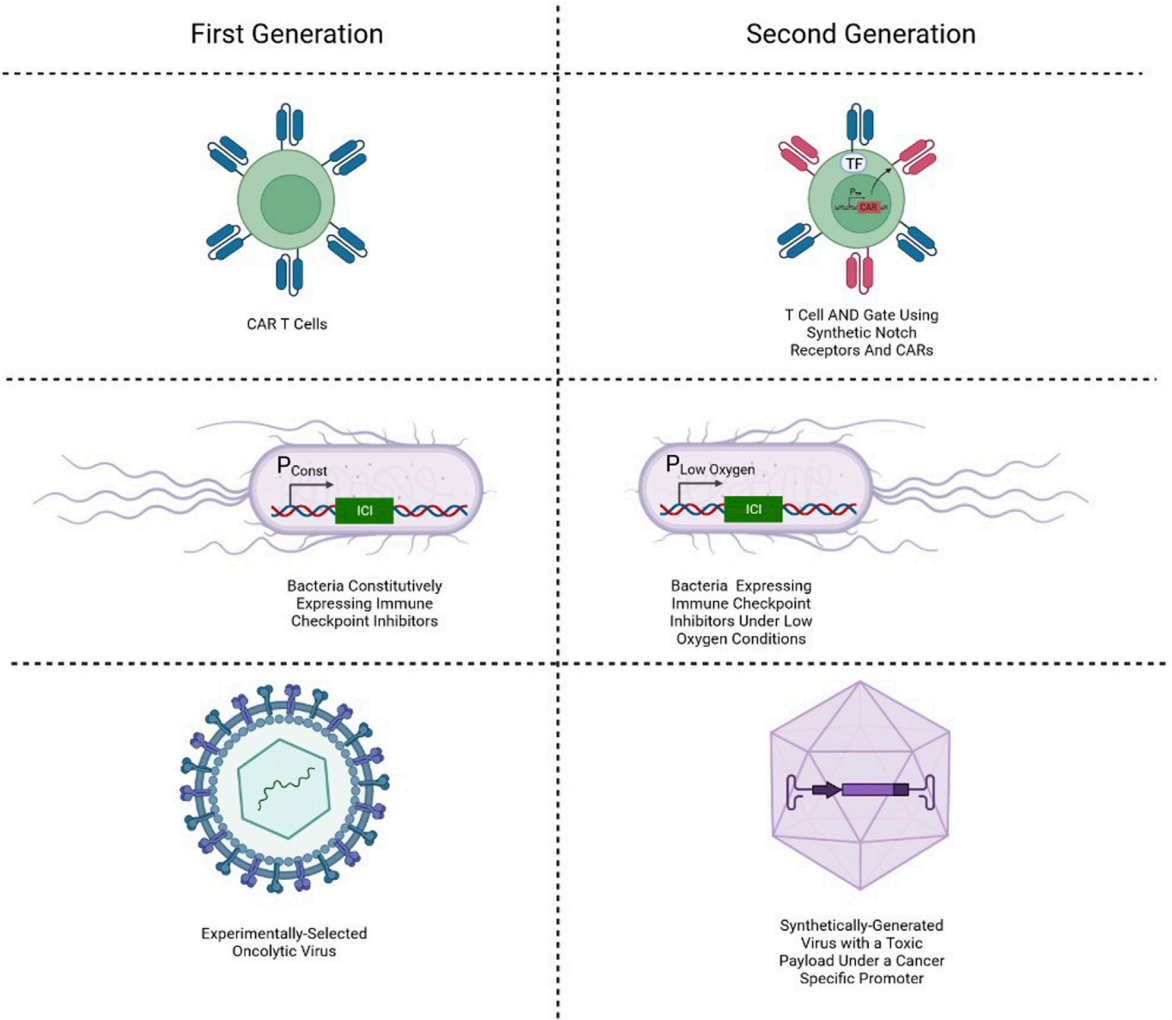
Simple single-input biological classifiers in cancer gene therapy, and their corresponding two-input AND gate classifiers.

CAR T cells, bacteriotherapy and OVs all rely on binary classification of the input (be it ligand, microenvironment, or proteome) to determine malignancy, and so essentially utilize simple biological digital computation. More recently, these digital computation solutions have been refined and improved, while maintaining a digital approach. Perhaps the most promising of those is the development of AND gate cancer classifiers (Figure 4). In the field of cell therapy, for example, an AND gate requiring two cancer-associated membranal markers has been constructed by utilizing synthetic notch (synNotch) receptors (Williams et al., 2020), a modified Notch-1 membranal receptor which allows the user to express premediated genes upon ligand binding. By designing a synNotch receptor to detect one marker and in turn activate the expression of a CAR targeting a second marker, an AND-gate means of cytotoxic activation can be attained. In cancer bacteriotherapy, environmentally dependent promoters have been implemented to generate a reaction not only upon colonization, but also upon recognition of markers characteristic of the tumor microenvironment like low oxygen concentration (Mengesha et al., 2006). And in virotherapy, carefully chosen promoters have been used to not only achieve activation upon presence of appropriate transcription factor associated with cancerous cells, but also logic gates that enable more precise classification. By using viruses to insert two cancer-specific promoters (which are cancer-selective by being active only in cancerous cells) that express proteins that combine and become an activator to a third promoter with a therapeutic output downstream, an AND gate has been constructed (Nissim et al., 2017).

**FIGURE 5:**
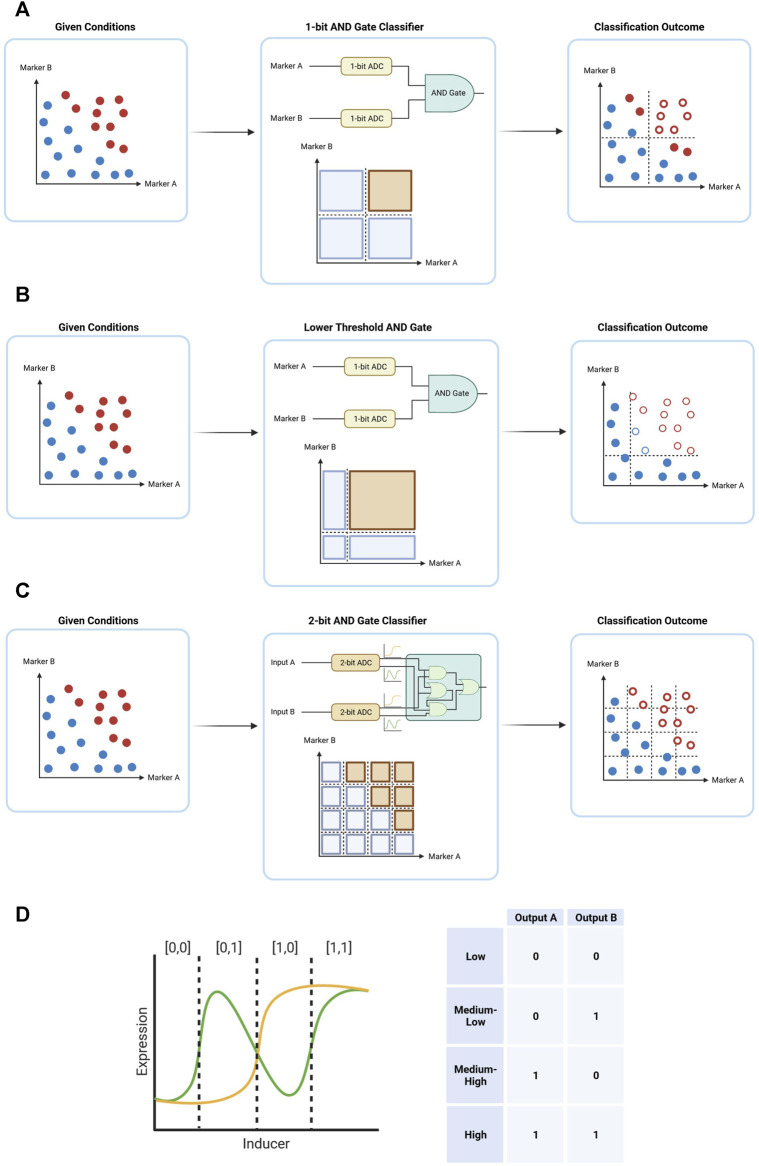
Simple forms of digital computation in cancer therapy, demonstrating how a classification system receives a system of cells with certain marker expression (blue–healthy cells, red–malignant cells) yields an outcome with certain of the cells eliminated (hollow circles). **(A)** A 2-input 1-bit AND gate. **(B)** A 2-input 1-bit AND gate with lower threshold for both inputs. **(C)** A schematic output of a 2-bit ADC, converting an analog input into a 2-bit output. **(D)** A 2-input 2-bit classification system. By further dividing the classification space, a finer differentiation in inputs can be achieved, resulting in an output with both higher specificity and higher sensitivity.

Despite the benefits of digital computation for cancer therapy, there are certain downsides to the use of this method. While proving a stable decision-making form of computation, a 1-bit digital system may not suffice in the classification of cells to benign and malignant. The tumor is a heterogenous environment with cells with varying phenotypes, and so often cannot be boxed into a simple AND classification without missing a substantial portion of the malignant cells (Figure 5A). And by simply decreasing the threshold between 0 and 1 in a 1-bit classification, there is also an increase in likelihood of damage to healthy cells, especially in the range close to the new lower threshold (Figure 5B). Therefore, a finer division of the input space is required—an approach with a higher resolution. An approach of this nature is possible by dividing each input further, by switching to a 2-bit analog to digital conversion (ADC). The 1-bit ADC is usually implemented through an inducer-dependent promoter, acting as a switch between 0 and 1 according to the inducer concentration. This promoter would sometimes express a transcription factor, which can then continue downstream to perform different interactions in the genetic circuit. A 2-bit ADC, on the other hand, would require the expression of two distinct transcription factors under the control of the same inducer. This type of ADC is accomplished by expressing two transcription factors, which together are able to attain all four forms of outputs expected from a 2-bit system—[0,0], [0,1], [1,0], [1,1] (Figure 5C). By switching from a 1-bit ADC to a 2-bit ADC, the increase in resolution theoretically allows finer decision making and thus an increase in specificity and sensitivity (Figure 5D). However, with an increase in the number of “bits” in the classifier there is an increase in the number of transcription factors needed to differentiate between different “bits”. A 2-input 1-bit AND gate utilizes just two transcription factors—one for each input. A 2-input 2-bit AND gate, on the other hand, utilizes four. And a 2-input 3-bit AND gate utilizes 6, pushing the limits of practical implementation. Hence, although biological digital circuits are generally easier to design than biological analog circuits, they often require a substantially larger number of parts to generate complex functions when compared to analog circuits. Ideally, future cancer gene therapies would build upon genetic circuits that merge the benefits of digital and analog biological computation.

## Neuromorphic computation in living cells

**FIGURE 6:**
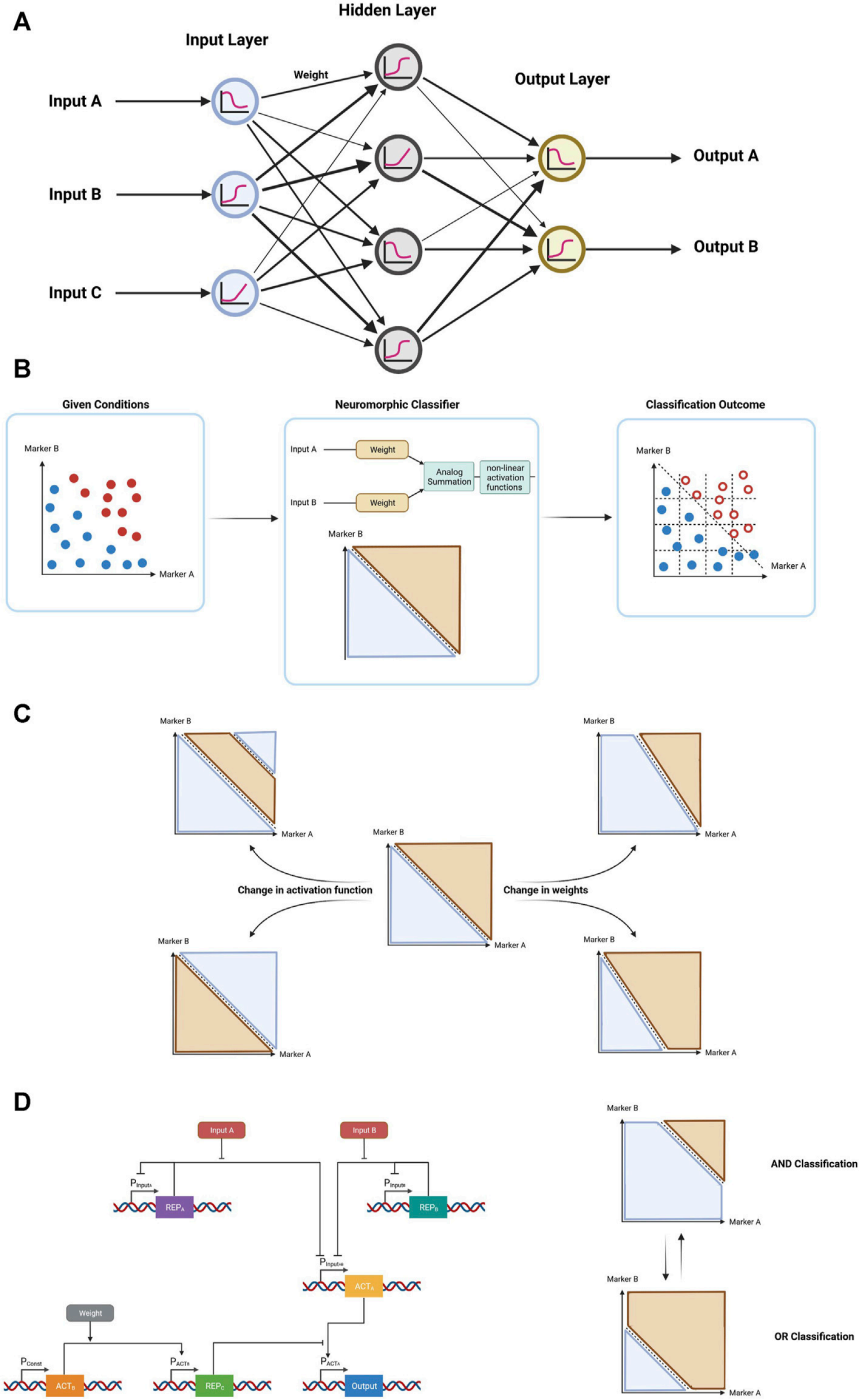
Neuromorphic computation and its advantages. **(A)** Representative schematic architecture of an artificial neural network with an input layer with 3 nodes, single hidden layer with 4 nodes, and output layer with 2 nodes **(B)** General form of neuromorphic computation demonstrating how a neuromorphic classification system receives a system of cells with certain marker expression (blue–healthy cells, red–malignant cells) yields an outcome with certain of the cells eliminated (hollow circles). **(C)** Through changes in the user-defined activation function and weights, different forms of classification can be easily achieved in neuromorphic computation. **(D)** Schematic of a gene circuit demonstrating a simple 2-input 1-weight neuromorphic classifier, allowing the user to define the function of the classification, choosing between an AND gate, and OR gate, or a midway point.

Fortunately, neuromorphic computation, a more recent form of biological computation, incorporates many of the advantages of both the digital and analog approaches. Neuromorphic computation employs design principles of neuronal systems and has been successfully utilized in a wide range of fields, chiefly software algorithms (Haykin, 1998) and electronics (Neftci et al., 2013; Wang et al., 2018; 2019). This form of computation can more efficiently perform certain tasks (e.g., pattern recognition, optimization), while often requiring significantly fewer parts, compared to the digital counterpart. Artificial neural networks are made out of perceptrons which consist of a linear combination of weighted analog input signals (Figure 6A). Biological neuromorphic computation systems have been implemented using different versions of the perceptron. Specifically, inside bacterial cells, the log-based versions of perceptrons have allowed efficient implementation of neuromorphic computation circuits. These types of perceptrons have a logarithmic input–output operation—making them suitable for the logarithmic nature of biochemical reactions—and implement a logarithmic classifier that partitions all input values into two classes of output data points—reliably generating either one of two states, similar to digital biological computation. Thus, neuromorphic computation combines analog information processing with digital decision-making capabilities using non-linear activation functions (e.g., sigmoid, rectifiers, step function) (Rizik et al., 2022). In doing so, we combine the stability and decision-making capabilities of digital computation with the low number of parts and natural functions of analog computation (Figure 6B). Furthermore, neuromorphic computation is highly adaptive—the weights can be easily changed to fit changes in circumstances and the activation function can be chosen according to the classification method required (Figure 6C). For example, a gene network with two inputs has been built where its functionality can be programmed from an AND to an OR logic gate through a change in a single parameter in the circuit (Rizik et al., 2022); (Figure 6D). In this example, the weight was determined by protein–protein interactions resulting in a new Hill-coefficient value. Cooperativity of transcription factors plays a critical role in determining the sensitivity of genetic regulatory circuits and synthetic genetic circuits (Danielli et al., 2020), and plays major role in neuromorphic computation.

Although the neuromorphic approach has only been developed recently, a few different methods of implementations have already been demonstrated. In one example, the potential of metabolism to perform analog computations and a sum classification has been investigated using synthetic metabolic circuits (Pandi et al., 2019). This investigation illustrated that, unlike a comparable digital biological system which would have needed multi-layered logic circuits, the tested metabolic adder was a simple one-layered device with fast execution times. Another design utilized protein heterodimers and engineered viral proteases to implement a synthetic protein circuit that performs winner-take-all neural network computation (Chen et al., 2022). This design demonstrated that a neuromorphic classification system can be even implemented using protein-level interaction—a useful type of architecture when constructing a system to react quickly to changes in the environment. Pattern recognition has been carried out with neuromorphic computation as well, where receiver bacteria collectively interacted with sender bacteria to generate decision-making through quorum sensing (Li et al., 2021). Chemical inducers were used to create 3 × 3 input patterns, which through gradient descent and suitable adjustment of weights (in this case the level of signal production by the sender cells) enable recognition of the pattern. Though all valuable applications, neuromorphic computation provides another major tool for cancer therapy—majority classification.

## Majority classification in gene therapy

**FIGURE 7:**
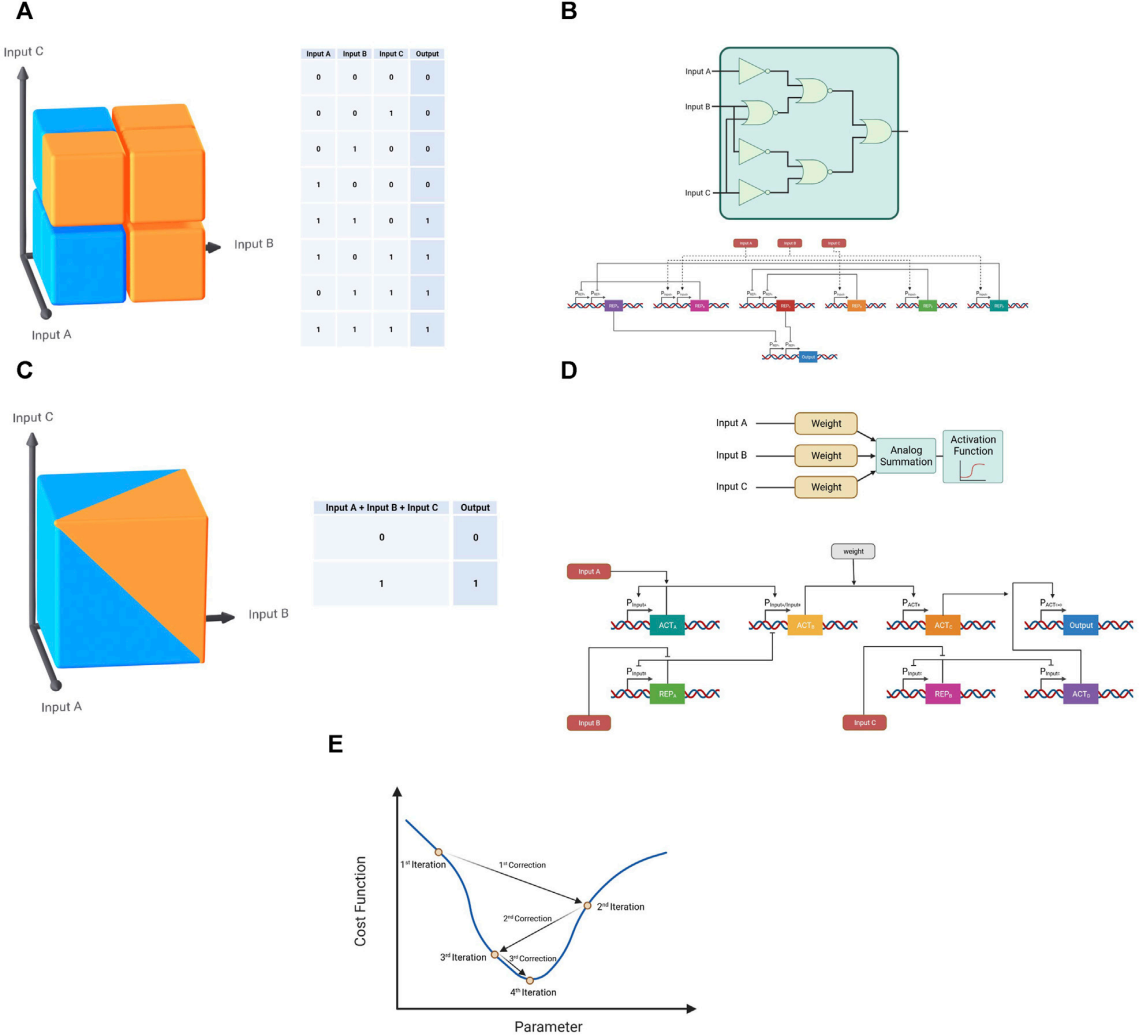
Majority classifications in digital and neuromorphic forms. **(A)** The classification of a digital majority classifier in three-dimensional space (Blue—0, Orange—1). **(B)** Schematic of a gene circuit demonstrating a digital majority classifier. **(C)** The classification of a neuromorphic majority classifier in three-dimensional space (Blue—0, Orange—1). **(D)** Schematic of a gene circuit demonstrating a neuromorphic majority classifier. **(E)** Representative demonstration of a gradient descent algorithm with the aim of reaching the minimum value of a cost function.

As mentioned previously, tumor heterogeneity poses a major hurdle for current cancer gene therapies. While increasing the resolution of 2-input systems is one approach to a solution, a different approach is the majority classification. Instead of increasing the resolution of discrepancy within each input, majority classification relies on inspecting 3 or more inputs–or in the case of cancer therapy, cancer markers (Figure 7). In the case of a 3-inputs system, a digital majority classifier would require at least 2 out of the 3 inputs to be positive in order to generate a positive output (Figure 7A). In cancer therapy, this would translate to an exceptional balance between specificity and sensitivity—a majority classification both requires at least two cancer-specific markers to be present (necessitating better specificity than a single input classification) and allows the absence of any one of the three inputs at any single moment without hindering its recognition abilities. Although a remarkable solution, to date few demonstrations in this form have been presented (Nielsen et al., 2016); (Figure 7B). One possible explanation for this lack of examples could be practical limitation. Similar to an increase in bits in an AND classification, an increase in the number of inputs and an increase in the complexity of the circuit to allow flexibility in its classification increase the number of orthogonal parts necessary to differentiate signals from different sources.

Fortunately, neuromorphic computation presents a novel solution to this limitation as well. By performing the summation in the analog domain before performing the classification through a sigmoid activation function, the neuromorphic system reduces the number of necessary parts and allows for simpler adjustment for different circumstances through the readjustment of the input weights (Figures 7C, D). This approach has been carried out successfully in bacteria in a non-medical application (Rizik et al., 2022), though there are no theoretical limitation to its use in mammalian cells, bacteria or viruses in a cancer therapy application. In this implementation optimization was necessary to achieve proper majority classification. Although 7 parts (promoter-gene pairs) were used, only two parameters were chosen to be optimized—the weight molecule concentration and the activity of the Plux promoter with six different values for the former and four for the latter. When taken into account with the different states for each one of the three inputs 192 samples would have been needed to conventionally find the optimal values. In order to optimize circuits of this complexity and reach the desired classification, artificial intelligence algorithms such as gradient descent can be performed, based on collected experimental biological data and repeated build-test-learn-correct cycles. Gradient descent is an algorithm used to efficiently reach optimal values defined as the minimum point of a certain cost function—a function used to quantify the performance of the system. In this algorithm, the “slope” of the function at a given point is used to deduce the direction of the minimal value (Figure 7E). This approach enables rapid circuit optimization, as was demonstrated in this majority classification implementation, which through gradient descent required only 48 experiments.

## Conclusion

Computation in current cancer gene therapies almost completely relies on digital design. Although this model has proven useful, both in its basic form and its more refined ones, it is clear that, moving forward, a different approach is required in order to push treatment forward and improve the cancer therapies—both in safety and in effectiveness. In this review, we presented the state-of-the-art solutions given through a digital approach, as well as their critical limitations. For that reason, we also introduced a probable solution to these limitations—a switch to a neuromorphic design. By taking advantage of the benefits of analog computation and attaching them to the functionality of digital computation, we believe that a superior form of therapeutic computation can be achieved. While further investigations into the use of neuromorphic computation in cancer therapy are necessary, we are confident in the current results presented by previous research in the field, and hope to witness a growth in exploration in this uncharted passage forward.
